# Color vision models: Some simulations, a general *n*‐dimensional model, and the *colourvision* R package

**DOI:** 10.1002/ece3.4288

**Published:** 2018-07-22

**Authors:** Felipe M. Gawryszewski

**Affiliations:** ^1^ Departamento de Zoologia Instituto de Ciências Biológicas Universidade de Brasília Brasília Brazil

**Keywords:** chromaticity diagram, color hexagon, color space, color triangle, color vision model, receptor noise‐limited model

## Abstract

The development of color vision models has allowed the appraisal of color vision independent of the human experience. These models are now widely used in ecology and evolution studies. However, in common scenarios of color measurement, color vision models may generate spurious results. Here I present a guide to color vision modeling (Chittka (1992, *Journal of Comparative Physiology A*, 170, 545) color hexagon, Endler & Mielke (2005, *Journal Of The Linnean Society*, 86, 405) model, and the linear and log‐linear receptor noise limited models (Vorobyev & Osorio 1998, *Proceedings of the Royal Society B*, 265, 351; Vorobyev et al. 1998, *Journal of Comparative Physiology A*, 183, 621)) using a series of simulations, present a unified framework that extends and generalize current models, and provide an R package to facilitate the use of color vision models. When the specific requirements of each model are met, between‐model results are qualitatively and quantitatively similar. However, under many common scenarios of color measurements, models may generate spurious values. For instance, models that log‐transform data and use relative photoreceptor outputs are prone to generate spurious outputs when the stimulus photon catch is smaller than the background photon catch; and models may generate unrealistic predictions when the background is chromatic (e.g. leaf reflectance) and the stimulus is an achromatic low reflectance spectrum. Nonetheless, despite differences, all three models are founded on a similar set of assumptions. Based on that, I provide a new formulation that accommodates and extends models to any number of photoreceptor types, offers flexibility to build user‐defined models, and allows users to easily adjust chromaticity diagram sizes to account for changes when using different number of photoreceptors.

## 1 INTRODUCTION

The study of proximate and ultimate causes of animal coloration has played a significant role in our understanding of evolutionary processes (for a review on the study of coloration see Cuthill et al., 2017). To study the selective forces acting on an organism coloration, it is crucial to understand how color patches are perceived by potential selective agents (e.g. a predator; Endler, 1990). Furthermore, animal vision may also evolve in response to environmental conditions, as is suggested by the correlation between light conditions and peak wavelength sensitivities of marine mammal photoreceptors (Fasick & Robinson, 2000).

There are several axes of variation in animal vision, such as density and distribution of receptors in the retina (Hart, 2001), eye resolution (Land & Nilsson, 2012), and presence of oil‐droplets in photoreceptors cells (Hart, Partridge, Bennett, & Cuthill, 2000). In terms of color vision, the most obvious differences are found in the spectral sensitivity of photoreceptors cells (Kelber, Vorobyev, & Osorio, 2003; Osorio & Vorobyev, 2008). For instance, most nonmammal vertebrates are tetrachromats, most insects are trichromats, and, contrary to mammals, both have a color perception that spans into the ultraviolet (Bowmaker, 1998; Briscoe & Chittka, 2001; Kelber et al., 2003; Osorio & Vorobyev, 2008). A fascinating illustration of how photoreceptor sensitivity may affect perceptual differences comes from human subjects that have undergone cataract treatment. The sensitivity curve of the human blue photoreceptor spans into the ultraviolet (UV) range, but humans are UV‐insensitive because pigments in crystallin filter out wavelengths below 400 nm. Cataract surgery occasionally replaces the crystallin with a UV‐transmitting lens, and anecdotal evidence suggests that those individuals are then able to see the world differently: new patterns are observed in flower petals, some garments originally perceived as black appear purple, and black light is perceived as blue light (Cornell, 2011; Stark & Tan, 1982).

Thus, studies of animal coloration can clearly benefit from appraisals of how color patches are perceived by nonhuman observers. Moreover, the same color patch may be perceived differently depending not only on the observer, but also on the conditions of exposure of the color patch (e.g. background color and environmental light conditions; Endler, 1990). With the advent of affordable spectrometers for reflectance measurements, application of color vision models became commonplace in the ecology and evolution subfields (Kemp et al., 2015). Together, some of the most important color vision papers have been cited over 2,800 times (Endler, 1990 (919); Vorobyev & Osorio, 1998 (601), Vorobyev, Osorio, Bennett, Marshall, & Cuthill, 1998 (460); Chittka, 1992 (324); Chittka, Beier, Hertel, Steinmann, & Menzel, 1992 (128); Endler & Mielke, 2005 (445); Google Scholar search on October 31st 2016).

Knowledge of model strength and limitations is crucial to assure reproducible and meaningful results from model applications. Thus, the motivation of this study is twofold: firstly, to compare and illustrate the consistency of between‐model results in common scenarios of color measurements; and secondly, to facilitate the use of color vision models by evolutionary biologists and ecologists by giving a unified framework which extends and generalizes the most commonly used color vision models. I did not aim to give an in‐depth analysis of the physiology of color vision, but rather, to provide a practical guide to the use of color vision models and to demonstrate their limitations and strengths so that users avoid the common pitfalls of color vision modeling. Guidance on other aspects of color vision models can be found elsewhere (Bitton, Janisse, & Doucet, 2017; Endler & Mielke, 2005; Hempel de Ibarra, Vorobyev, & Menzel, 2014; Kelber et al., 2003; Kemp et al., 2015; Lind & Kelber, 2009; Olsson, Lind, & Kelber, 2017a,2017b; Osorio & Vorobyev, 2008; Renoult, Kelber, & Schaefer, 2017; Vorobyev, Osorio, Peitsch, Laughlin, & Menzel, 2001; White, Dalrymple, Noble, & O'Hanlon, 2015).

## 2 GUIDELINES AND LIMITATIONS

As any model, color vision models are simplified representations of reality. Their mathematical formulation imposes limits to their predictive power. Many of these limitations have been pointed out by several authors (Bitton et al., 2017; Lind & Kelber, 2009; Vorobyev, 1999; Vorobyev & Brandt, 1997; Vorobyev, Hempel de Ibarra, Brandt, & Giurfa, 1999). Nonetheless, as stressed recently, several papers in the evolution and ecology subfields still misuse color vision models (Marshall, 2017; Olsson et al., 2017b). Some of these limitations may be obvious to scientists working directly on color vision, but are not by many nonspecialists that apply color vision models to their research. In this section, I compile and illustrate those limitations by a series of simulations. My focus is on the limitations arising from the mathematical formulation of each model, which are often obscure to many nonspecialists in the field.

I modeled the perception of the honeybee (*Apis mellifera*) using the following color vision models: Chittka (1992) color hexagon model (hereafter CH model), Endler and Mielke (2005) model (hereafter EM model), and linear and log‐linear versions of the receptor noise model (hereafter linear‐RNL and log‐RNL models (Vorobyev et al., 1998; Vorobyev & Osorio, 1998). I began with a basic model setup using simulated data. I then proceeded to make a series of changes to this basic model to illustrate how models behave with typical input data used in ecology and evolution papers. At the end, I used real flower reflectance data to compare model results. I violated some model assumptions, for example, I applied the linear‐RNL model to nonsimilar colors, so that model behaviour could be visualized under suboptimum conditions.

Human color perception can be divided into two components: the chromatic (hue and saturation) and achromatic (brightness/intensity) dimensions. These models are representations of the chromatic component of color vision (Renoult et al., 2017). Color vision models are based on photoreceptor photon catches of each photoreceptor type in the retina. Photon catches depend on the illuminant spectrum reaching the observed object, the reflectance of the observed object, the sensitivity curve of photoreceptors, and the background reflectance (for details see Supporting Information Appendix S1).

These models assume that color vision is achieved by neural opponency mechanisms, which is supported by experimental data (Kelber et al., 2003; Kemp et al., 2015, but see Thoen, How, Chiou, & Marshall, 2014 for an exception to this rule). The exact opponent channels are usually not known (Kelber et al., 2003; Kemp et al., 2015), but empirical studies suggest that the exact opponency channels do not need to be known for a good prediction of behavioural responses by color vision models (Cazetta, Schaefer, & Galetti, 2009; Chittka et al., 1992; Spaethe, Tautz, & Chittka, 2001; Vorobyev & Osorio, 1998). Taking that into account, the three models presented here assume that inputs from photoreceptors are weighted equally and are all opposed against each other.

In addition, photoreceptor values are adjusted taking into consideration the photon catch arising from the environment background (Supporting Information Appendix S1, Equation S3), which tries to emulate the physiological adaptation of photoreceptors to the environmental light conditions and the color constancy (Chittka, Faruq, Skorupski, & Werner, 2014). Photon catches relative to the background are then transformed to represent the relationship between photoreceptor input and output. Each model will apply a different transformation (e.g. identity, ln, hyperbolic; for details see Supporting Information Appendix S1), but the rationale behind all these are the nonlinear relationship between photoreceptor input and output. However, only the EM (Endler & Mielke, 2005) and log‐RNL models (Vorobyev et al., 1998) apply the natural logarithm as formulated by the Fechner‐Weber law of psychophysics. The CH model applies a hyperbolic transformation that also simulates a nonlinear relationship between photoreceptor input and output, and the linear RNL models is to be applied only to similar colors so that the relationship is nearly linear (Vorobyev & Osorio, 1998). Furthermore, EM model uses only relative photoreceptor output values (sum of photoreceptor values equal 1), not their absolute values, which is based on the biological observation that only relative differences in photoreceptor outputs are used in a color opponency mechanisms (Endler & Mielke, 2005).

### 2.1 First simulation: basic setup

I used honeybee worker (*A. mellifera*) photoreceptor sensitivity curves (data from Peitsch et al., 1992 available in Chittka & Kevan, 2005; Supporting Information Figure S1a). For the background reflectance spectrum, I created a theoretical achromatic reflectance with a constant 7% reflectance across 300–700 nm (Supporting Information Figure S1b). For the illuminant, I used the CIE D65, a reference illuminant that corresponds to midday open‐air conditions (Supporting Information Figure S1c). In addition, RNL models assume that, under bright light conditions, color discrimination threshold is limited by photoreceptor noise (Vorobyev & Osorio, 1998; for dim light conditions shot noise also limits discrimination; Vorobyev et al., 1998; see Olsson et al., 2017a for a recent review). For these models, I used measurements of honeybee photoreceptor noise (0.13, 0.06 and 0.12 for short‐, medium‐, and long‐wavelength photoreceptors; data from Peitsch, 1992 available in Vorobyev & Brandt, 1997). With respect to the stimulus reflectance spectra, I generated reflectance curves using a logistic function (see Supporting Information Appendix S1 for details). I generated curves with reflectance values from of 10% to 60%, and midpoints varying from 300 to 700 nm with 5 nm intervals, in a total of 81 reflectance spectra (Supporting Information Figure S1d). For each model, I calculated photoreceptor outputs, color loci (*x* and *y*), and the chromatic distance to the background (Δ*S*) for each reflectance spectra using equations for CH, EM, and RNL models (Equations S2–S19 see Supporting Information Appendix S1 for details on model calculations). To illustrate the generality of the results from these simulations, I ran the same simulations with a Gaussian function to generate the stimulus reflectance spectra, and for tetrachromatic avian vision (see Supporting Information Appendix S1 for methods and results; results are qualitatively very similar to the original simulations).

**Figure 1:**
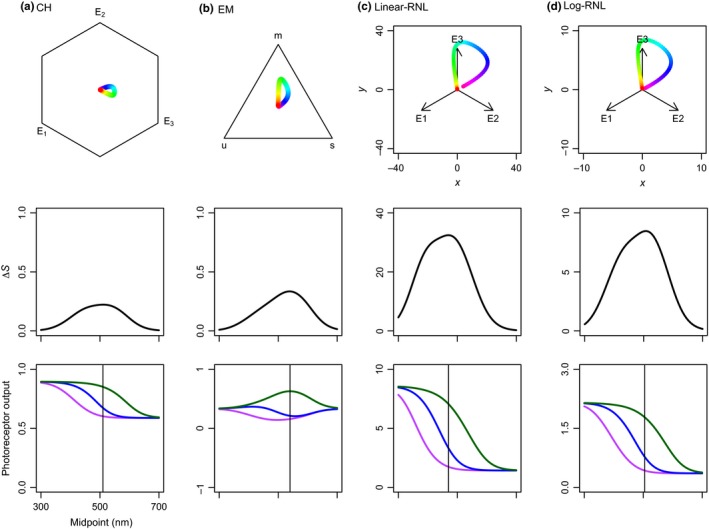
Chromaticity diagrams, Δ*S*, and photoreceptor outputs of the basic setup of color vision model simulations: (a) Chittka (1992) color hexagon (CH), (b) Endler and Mielke (2005) color triangle (EM), and (c) linear and (d) log‐linear Receptor Noise Limited models (Linear‐RNL and Log‐RNL; Vorobyev & Osorio, 1998; Vorobyev et al., 1998). Colors in chromaticity diagrams correspond to reflectance spectra from Supporting Information Figure S1d. Δ*S*‐values (middle row) and photoreceptor outputs (bottom row) as a function of reflectance spectra with midpoints from 300 to 700 nm. Violet, blue, and green colors represent short, middle, and long *λ*_max_ photoreceptor types, respectively. Vertical lines represent the midpoint of maximum Δ*S*‐values

In this first setup, models are congruent with respect to their results. The chromaticity diagrams indicate a similar relative position of reflectance spectra between models (Figure 1). All of them estimate a bell‐shaped Δ*S* curve, with maximum values around a 500‐nm midpoint wavelength (Figure 1).

### 2.2 Second simulation: stimulus reflectance lower than background reflectance

In the second simulation, I removed 10 percentage points to the stimulus reflectance spectra (Supporting Information Figure S2a). In this case, my aim was to (a) analyze how a relatively small change in reflectance curves affect model results, as small changes in overall reflectance values may be an artifact of spectrometric measurement error (for guidance on spectrometric reflectance measurements, see Anderson & Prager, 2006); and (b) to create reflectance spectra that would generate a lower photoreceptor response from stimulus than the background.

**Figure 2:**
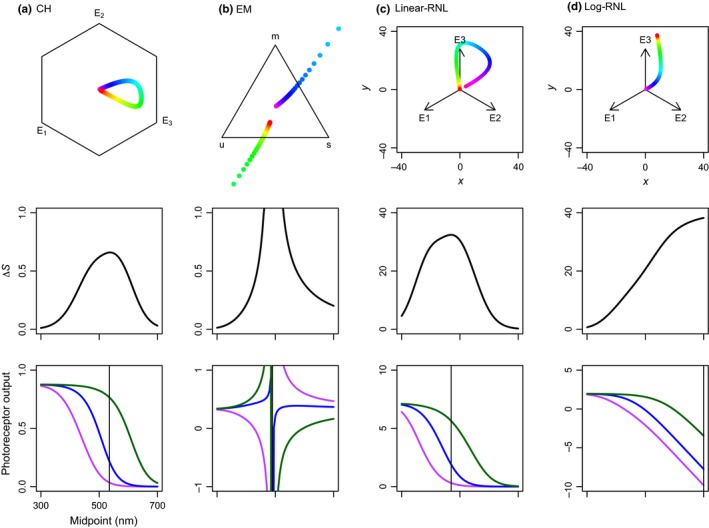
Chromaticity diagrams, Δ*S*, and photoreceptor outputs of the second simulation—10 percentage points removed from reflectance values: (a) Chittka (1992) color hexagon (CH), (b) Endler and Mielke (2005) color triangle (EM), and (c) linear and (d) log‐linear Receptor Noise Limited models (Linear‐RNL and Log‐RNL; Vorobyev & Osorio, 1998; Vorobyev et al., 1998). Colors in chromaticity diagrams correspond to reflectance spectra from Supporting Information Figure S2d. Δ*S*‐values (middle row) and photoreceptor outputs (bottom row) as a function of reflectance spectra with midpoints from 300 to 700 nm. Violet, blue, and green colors represent short, middle, and long *λ*_max_ photoreceptor types, respectively. Vertical lines represent the midpoint of maximum ΔS‐values

In this simulation, results projected into chromaticity diagrams show differences between model predictions of color perception for the same reflectance spectrum (Figure 2). Contrary to the first simulation, in the EM model, points follow two lines increasing in opposite directions, with data points reaching values outside color space limits (Figure 2b). The EM model estimates spurious Δ*S* values for reflectance curves with midpoints between 450 and 550 nm (Figure 2b). A maximum Δ*S* of 116 is reached at 490 nm midpoint wavelength; however, by the model definition, the Δ*S* maximum value is 0.75 (Endler & Mielke, 2005). Photoreceptor outputs also reach nonsensical negative values and values above 1 (by model definition, maximum photoreceptor output should vary between 0.0 and 1.0; Figure 2b). This happens when relative photon catches (*q*_i_; Supporting Information Appendix S1Equation S4, ) are below 1 (i.e. background photon catch is higher than stimulus photon catch), and therefore, the ln‐transformation generates negative values. Consequently, the denominator in Supporting Information Equation S9 may reach values close to zero, which causes photoreceptor outputs to tend to infinity.

Comparable to the EM model, the log‐RNL model generates nonsensical negative photoreceptor excitation values (Figure 2d). Again, this happens because when the relative photoreceptor photon catch (*q*_i_; Supporting Information Appendix S1Equation S4, ) is below 1, the ln‐transformation generates negative values (Supporting Information Appendix S1Equation S15, ). Consequently, this model now presents a sigmoid Δ*S*, increasing from short to long midpoint wavelengths (maximum Δ*S* at 700 nm; Figure 2d).

Therefore, color vision models, especially those that are log‐transformed (EM model and log‐RNL model) and convert photoreceptor output to relative values (EM model), are prone to produce nonsensical results when the observed reflectance generates a lower response than the background (*Q*_i _< *Q*_Bi_; Equation S4, Supporting Information Appendix S1).

The transformation of photoreceptor inputs also illustrates a common misconception related to the use of color vision models. Models are intended to be insensitive to variation in intensity only. Nonetheless, in practice, models are insensitive to variation in photoreceptor outputs as long as the difference between outputs remains the same. However, this does not mean that reflectance spectra that only differ in intensity will generate identical model outputs. For instance, a reflectance spectrum that generates photoreceptor outputs of *E*_1 _= 0.1, *E*_2_ = 0.2 and *E*_3_ = 0.3 will lie at the exact same color locus coordinates as another reflectance that generates photoreceptor outputs of *E*_1_ = 0.2, *E*_2_ = 0.3, and *E*_3_ = 0.4 because differences between photoreceptor outputs remain the same (i.e. *E*_3_ − *E*_1_ = 0.2; *E*_2_ − *E*_1_ = 0.1; and *E*_3_ − *E*_2_ = 0.1). Nonetheless, reflectance spectra that differ only in intensity (simulation 1 vs. simulation 2) will most likely generate distinct differences between photoreceptor outputs because of the photoreceptor transformation. Consequently, these spectra will lie at different positions in the animal color space (color locus coordinates; compare Figures 1 and 2). CH model, in special, predicts different color loci for reflectance curves that only differ in intensity due to the hyperbolic transformation (Chittka, 1992). There is a controversy whether this represents a biological phenomenon (Chittka, 1992, 1999) or it is a model limitation (Vorobyev et al., 1999).

### 2.3 Third simulation: achromatic stimulus and chromatic background

In the basic model, I used an achromatic reflectance spectrum (7% reflectance from 300 to 700 nm). In practice, however, most studies that apply color vision models use chromatic reflectance backgrounds, such as a leaf (e.g. Vorobyev et al., 1998), or an average of background‐material reflectance spectra (e.g. Gawryszewski & Motta, 2012). Models are constructed so that the background reflectance spectrum lies at the center of the color space. Vorobyev and Osorio (1998) specifically state that their linear receptor noise model is designed to predict perception of large targets, in bright light conditions and against an achromatic background. Despite this, given that photoreceptors adapt to the light environment condition, usage of chromatic background is probably reasonable (Vorobyev et al., 1998). I generated achromatic reflectance spectra ranging from 5% to 95% in 10 percent point intervals (Supporting Information Figure S2b), and instead of having an achromatic background, I used a chromatic background (Supporting Information Figurre S2c). The background is the average reflectance of leaves, leaf litter, tree bark, and twigs collected in an area of savanna vegetation in Brazil (data from Gawryszewski & Motta, 2012).

**Figure 3:**
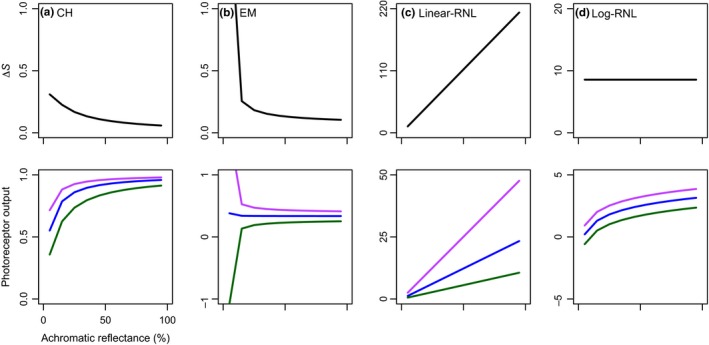
Third setup of color vision model simulations—achromatic stimulus against chromatic background: (a) Chittka (1992) color hexagon (CH), (b) Endler and Mielke (2005) color triangle (EM), and (c) linear and (d) log‐linear Receptor Noise Limited models (Linear‐RNL and Log‐RNL; Vorobyev & Osorio, 1998; Vorobyev et al., 1998). Δ*S*‐values (top row) and photoreceptor outputs (bottom row) as a function of spectra with achromatic reflectance from 5% to 95%. Violet, blue, and green colors represent short, middle, and long *λ*_max_ photoreceptor types, respectively

The chromatic background causes differences in background photoreceptor photon catches. Consequently, achromatic reflectance spectra do not lie at the center of the color spaces as would be expected. The CH model shows a maximum Δ*S* value of 0.31 at 5% reflectance achromatic spectrum (Figure 3a). Δ*S* values then decrease as the reflectance value of achromatic spectra increases (Figure 3a). The EM model produces spurious values at 5% reflectance achromatic spectrum because it generates negative photoreceptor output values (Figure 3b). From 15% and beyond, Δ*S* values then decrease as the reflectance value of achromatic spectra increases (Figure 3b). The linear‐RNL model shows a linear increase in Δ*S* values as the reflectance value of achromatic spectra increases (Figure 3c). Similarly, photoreceptor outputs also increase linearly as the reflectance value of achromatic spectra increases, but with different slopes for each photoreceptor type (Figure 3c). Contrary to other models, Δ*S*‐values in the log‐RNL model do not change with varying reflectance values of achromatic spectra (Figure 3d). Although photoreceptor outputs increase as reflectance value of achromatic spectra increases (Figure 3d), the difference between photoreceptor outputs remains the same. Consequently, Δ*S*‐values do not change.

This simulation shows that CH and EM models predict counterintuitive values because a highly reflective achromatic stimulus is predicted to have a lower Δ*S*‐value than a spectrum with similar reflectance to the background. This phenomenon has already been discussed previously both theoretically and experimentally (Stoddard & Prum, 2008; Vorobyev, 1999; Vorobyev & Brandt, 1997; Vorobyev et al., 1999). For instance, in a laboratory experimental setup, Vorobyev et al. (1999) showed that bees made more mistakes when trying to detect a grey target against a green background than when trying to detect a white target again the same green background.

Another common misconception arises from the use of detectability/discriminability thresholds. The RNL model, for instance, is a good predictor of the detectability of monochromatic light against a gray background (Vorobyev & Osorio, 1998). For this model, and given the experimental condition, a Δ*S *=* *1 equals one unit of just noticeable difference (JND; Vorobyev & Osorio, 1998). However, this threshold is not fixed. For zebra finches, for instance, the same pair of similar red objects have a discriminability threshold of ca. 1 JND when the background is red, but much higher when the background is green (Lind, 2016). Furthermore, the relationship between ΔS values and probability of discriminability varies between species and it is not necessarily linear, in particular for ΔS values that greatly surpass threshold values (Garcia, Spaethe, & Dyer, 2017). In addition, correct model parametrization is vital for RNL models, which are very sensitive to correct photoreceptor noise values (Lind & Kelber, 2009; Olsson et al., 2017a) and the relative abundance of photoreceptors in the retina (Bitton et al., 2017).

### 2.4 Real reflectance data: comparison between models

In this setup, my aim was to compare model results using real reflectance data. I used 858 reflectance spectra from flower parts collected worldwide and deposited in the Flower Reflectance Database (FReD; Arnold, Faruq, Savolainen, McOwan, & Chittka, 2010). I used only spectrum data that had a wavelength range from 300 to 700 nm. Data were then interpolated to 1‐nm intervals and negative values converted to zero. I used the same reflectance background from simulation 03, and other model parameters identical to the basic model setup. I compared model results visually, and by testing the pairwise correlation between the model's Δ*S* values. I used the Spearman correlation coefficient because data did not fulfill assumptions for a parametric test.

When real flower reflectance spectra are used, models also give different relative perception for the same reflectance spectrum. The results of the CH model and the log‐RNL model are similar both qualitatively and quantitatively: color loci projected into the color space (Supporting Information Figure S3) show a similar relative position of reflectance spectra, and there is a high correlation score between Δ*S* values (*ρ* = 0.884; *N* = 858; *p* < 0.001). Even though many EM points lie outside the chromaticity diagram (Supporting Information Figure S3b), results suggest a high concordance between CH and EM models (*ρ* = 0.889; *N* = 858; *p* < 0.001). There was moderate concordance between the linear and log versions of the RNL model (*ρ* = 0.434; *N* = 858; *p* < 0.001) and between the EM and log‐RNL models (*ρ* = 0.662; *N* = 858; *p* < 0.001). Finally, there was poor concordance between the linear‐RNL model and both EM models (*ρ* = −0.264; *N* = 858; *p* < 0.001), as well as the linear‐RNL and CH models (*ρ* = 0.037; *N* = 858; *p* = 0.278).

In addition to the limitations commented above, these models also do not incorporate higher order cognition abilities that may affect how color is perceived (Dyer, 2012). In bees, for instance, previous experience, learning and experimental conditions may affect their behavioural discriminability thresholds (Chittka, Dyer, Bock, & Dornhaus, 2003; Dyer, 2012; Dyer & Chittka, 2004; Dyer, Paulk, & Reser, 2011; Giurfa, 2004), and in humans, the ability to discriminate between colors is affected by the existence of linguistic differences for colors (Winawer et al., 2007). Moreover, models presented here are pairwise comparisons between color patches, which do not incorporate the complexity of an animal color pattern composed of a mosaic of color patches of variable sizes. Endler and Mielke (2005) provide a methodological and statistical tool that can deal with a cloud of points representing an organism's color patches. Use of hyperspectral cameras or adapted DSLR cameras may facilitate the analysis of animal coloration as a whole (Chiao, Wickiser, Allen, Genter, & Hanlon, 2011; Stevens, Párraga, Cuthill, Partridge, & Troscianko, 2007). Other aspects that may be important when detecting a target, such as size, movement, light polarization (Cronin, Johnsen, Marshall, & Warrant, 2014), and color categorization (Hempel de Ibarra et al., 2014; Kelber & Osorio, 2010), are also not incorporated into those models.

Therefore, accurate application of color vision models depends on the inspection of photoreceptor output values, knowledge of model assumptions, comprehension of the mathematical formula used for constructing each model, and familiarity with mechanisms of color vision of the animal being modeled. Comparison of model results with field and laboratory‐based behavioural experiments are also crucial to complement and validate model results.

## 3 A GENERIC METHOD FOR *n*‐DIMENSIONAL MODELS

**Figure 4:**
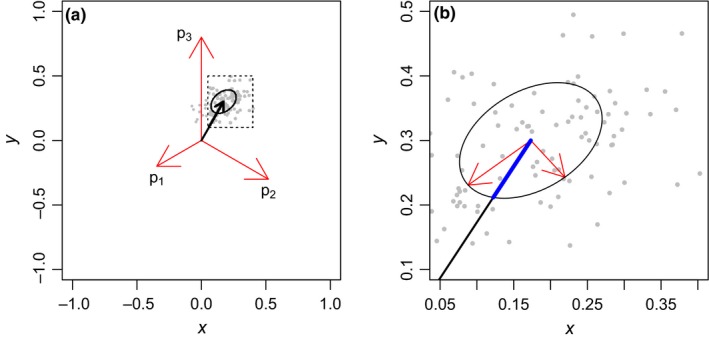
(a) Example of photoreceptor outputs (*p*) from a trichromatic animal projected as vectors (red arrows) into a chromaticity diagram. The black arrow denotes a vector ($\vec{s}$) resulting from adding vectors ${\vec{p}}_{1}$, ${\vec{p}}_{2}$, and ${\vec{p}}_{3}$. (Equation 6). Its components are the coordinates in the color space and its length, the ΔS value to the background in Chittka (1992) and Endler and Mielke (2005) models. Receptor noise models assume that discriminability thresholds are defined by noise at the photoreceptors. Gray points denote randomly generated vectors from normally distributed *p* values and their receptor noise (one standard deviation). Ellipse denotes the standard deviation. The ellipse is calculated from $\vec{p}$ vectors and their receptor noise. (b) Inset showing the ellipse and its eigenvectors, with the size adjusted to one standard deviation. The length of the line segment in blue represents vector $\vec{s}$ standard deviation. Receptor noise value against the background is simply the length of vector $\vec{s}$ divided by its standard deviation

Despite some differences between these models, they can all be understood using the same general formulae. As explained in the section above, color vision is achieved by neural opponency mechanisms (Kelber et al., 2003; Kemp et al., 2015), although for most species the opponency channels have not been identified (Kelber et al., 2003; Kemp et al., 2015). In practice, the solution is to build a model so that all photoreceptor outputs are compared simultaneously. This is achieved by projecting photoreceptor outputs as vectors (vector lengths represent output values) into a space so that all vectors have the same pairwise angle (i.e. the resulting vector has length zero when all vector lengths are equal; Figure 4). Each model will present differently arranged vectors. However, they can all be reduced to the same general formula because vector position in relation to the axes has no biological significance as long as they preserve the same pairwise angle (see Vorobyev & Osorio, 1998).

By adding vectors, the length of the resultant vector represents the chromaticity distance of the stimulus against the background, and vector components represent the stimulus coordinates in the color space (color locus; Chittka, 1992). Vorobyev and Osorio (1998) assume that noise at photoreceptors limits chromatic discrimination. In this case, each photoreceptor has a specific noise, and the chromaticity distance is given by the resultant photoreceptor length divided by its noise (see calculation below; Figure 4).

For a generic *n*‐dimensional method, let *i* be the number of photoreceptor sensitivity curves. Assuming an opponency mechanism, the animal chromaticity diagram will have *n = i* − 1 dimensions. In this space, there will be *i* vectors, each representing the output of one photoreceptor type (Figure 4a). Each vector will have *i* − 1 components (*n* = *i* − 1), each representing one coordinate in the chromaticity diagram (Figure 4a). Photoreceptors are assumed to be weighted equally and give sum zero; therefore, their pairwise angle is given by:

(1) $$\theta =\text{arccos}\left(-\frac{1}{n}\right).$$

Then, the last component of a generic unit vector (*v* = [*v*_1_, *v*_2_, *v*_3_ … *v*_n_]) projected into a chromaticity diagram with *n* = *i* − 1 dimensions can be found by the following equations:

(2) $$v_{\left[n\right]}=\text{cos}(\theta ).$$

If the chromaticity diagram has only one dimension, (*i* = 2), then the generic vector has only one component (*n* = 1), given by Equation (2). For a chromaticity diagram with more than one dimension (*i* > 2), other vector components are found by the following equation:

(3) $$v_{\left[n-k\right]}=-\frac{1}{n-k}\sqrt{1-\sum_{m\;=\;n-k+1}^{n}v_{m}^{2}}$$

where *n* is the total number of vector components, and *k* = (1, 2, 3, …, *n *− 1). Then a matrix of column vectors (size: *i *× *n*; each column represents one vector) with unit vectors equidistant from each other can be found by the following equation:

(4) $$V_{i\times n}=\left(\begin{array}{ccccccccccc} 1v_{1} & & -1v_{1} & & 0v_{1} & & 0v_{1} & & & & 0v_{1}\\ 1v_{2} & & 1v_{2} & & -2v_{2} & & 0v_{2} & & \ldots & & 0v_{2}\\ 1v_{3} & & 1v_{3} & & 1v_{3} & & -3v_{3} & & & & 0v_{3}\\ & & & & \vdots & & & & \ddots \\ 1v_{n} & & 1v_{n} & & 1v_{n} & & 1v_{n} & & & & -nv_{n} \end{array}\right)$$

where *v* is the generic unit vector, as found by Equations (1), (2), (3). Equations (1), (2), (3), (4) were found empirically.

Note that this is one of infinite possible solutions to project *n* vectors into a (*n* − 1) dimensional space. Although it will not have a biological meaning, nor affect results, other orientations of matrix *V* can be achieved by vector rotation matrices.

With matrix *V*, one can find a vector, whose components represent coordinates in the color space (*X*_1_, *X*_2_, …, *X*_n_), by multiplying matrix *V* by a column vector with photoreceptor output as its components (*p* = (*p*_1_, *p*_2_, *p*_3_, … *p*_i_)):

(5) $$V\vec{p}=\vec{x}$$

(6) $$\left(\begin{array}{cccccc} 1v_{1} & -1v_{1} & 0v_{1} & 0v_{1} & & 0v_{1}\\ 1v_{2} & 1v_{2} & -2v_{2} & 0v_{2} & \ldots & 0v_{2}\\ 1v_{3} & 1v_{3} & 1v_{3} & -3v_{3} & & 0v_{3}\\ & & \vdots & & \ddots \\ 1v_{n} & 1v_{n} & 1v_{n} & 1v_{n} & & -nv_{n} \end{array}\right)\left(\begin{array}{c} p_{1}\\ p_{2}\\ p_{3}\\ \vdots \\ p_{n} \end{array}\right)=\left(\begin{array}{c} X_{1}\\ X_{2}\\ \vdots \\ X_{n-1} \end{array}\right)$$

One may also represent Equation 6 as formulae, as is usually performed when presenting color vision models.

Matrix *V* can be manipulated to accommodate different color vision models. Chittka (1992) assumes a maximum vector length of one (Equation S5, Supporting Information Appendix S1); therefore, matrix *V* can be used directly. The tetrachromatic version of Endler and Mielke (2005), however, assumes a maximum length of 0.75. Therefore, matrix *V* must be multiplied by a scalar with the desired length (see Supporting Information Appendix S1 for detail on model calculation using formulae above).

In the original study, Vorobyev and Osorio (1998) provided a method to calculate chromaticity distances (Δ*S*) independently of the matrix *V*, and their method is already applicable to any number of photoreceptor types (see also Clark et al. 2017 to another model extension). However, within Vorobyev and Osorio (1998) formulation, it is possible to find a space representing RNL model color space in terms units of receptor noise (see for instance Renoult et al., 2017 and Pike, 2012a,2012b). For a 2‐D color space, the noise standard deviation will be given by the line segment, from the centre to the edge of the standard deviation contours, in the same direction as the vector representing the signal (Figure 4b). Then a vector, ($\vec{s}$), whose components represent coordinates in the color space, is found by dividing vector components by the length of the noise line segment (Figure 4b). This calculation can be performed by a simple change in Vorobyev and Osorio (1998, equation A7). In this new equation, the covariance matrix of receptor noise in coordinates of the V matrix (equation A6 in Vorobyev & Osorio, 1998) are square‐root transformed and multiplied by x so that vector length represents chromaticity distances instead of chromaticity distances to the square as in equation A7 (Vorobyev & Osorio, 1998):

(7) $$\vec{s}=\sqrt{{\left(VRV^{T}\right)}^{-1}}\vec{x}$$

where *V* is the matrix in Equation (4), *T* represents the transpose, $\vec{x}$ is a column vector with color locus coordinates (as in Equation 6), and *R* is a covariance matrix of photoreceptor output values. Since photoreceptor outputs are not correlated, *R* is a diagonal matrix with photoreceptor output variance (receptor noise) in their diagonal elements ($e_{i}^{2}$; Vorobyev & Osorio, 1998). The main advantage of Equation 7 is to allow visualization of RNL data into a space where distance between points corresponds to chromaticity distance values as calculated by Vorobyev and Osorio (1998) original equations.

The boundaries of the color space will depend on the calculation of photoreceptor outputs. For instance, in Chittka (1992) color hexagon model, a trichromatic color space is represented by a hexagon, whereas in the Endler and Mielke (2005) model, the color space is reduced to a triangle because summation of photoreceptor outputs is assumed to equal 1 (Equation S9; Supporting Information Appendix S1). In contrast, transformations used by receptor noise models (Vorobyev & Osorio, 1998; Vorobyev et al., 1998) impose no upper limit, and therefore, the color space has no defined boundary.

Furthermore, when extending models to accommodate different numbers of photoreceptors (e.g. from a tetrachromatic to a pentachromatic version), there is often a trade‐off between preserving the edge size (distance between color space vertices) and preserving vector length. Pike (2012a), for instance, holds edge distance constant when changing color space dimensions; however, this comes at the cost of increased vector length as the number of dimensions increases. In practice, preserving an edge length of $\sqrt{2}$ means that for a trichromat, the maximum chromaticity distance from the center to the edge, is 0.816, but 0.866 for a tetrachromat. In contrast, chromaticity distances in receptor noise limited models are independent of the color space geometry (Vorobyev & Osorio, 1998). The generic matrix *V* allows for a user‐defined adjustment of color space size.

Distances in chromaticity diagrams are assumed to represent chromaticity similarities between two colors. The assumption is that the longer the distance, the more dissimilar the two perceived colors are (note, however, that this relationship is not necessarily linear; see for instance Garcia et al., 2017). Chromaticity distances between a pair of reflectance spectra (*a* and *b*) are found by calculating the Euclidian distance between their color loci in the color space:

(8) $$\Delta S=\sqrt{{\left(X_{1_{a}}-X_{1_{b}}\right)}^{2}+{\left(X_{2_{a}}-X_{2_{b}}\right)}^{2}+\ldots +{\left(X_{n_{a}}-X_{n_{b}}\right)}^{2}.}$$

By definition, background reflectance lies at the centre of the background ($X_{1_{b}}=0,\;\;X_{2_{b}}=0,\;\ldots ,X_{n_{b}}=0)$.

## 4 COLOURVISION: R PACKAGE FOR COLOR VISION MODELS AND RELATED FUNCTIONS

**Figure 5:**
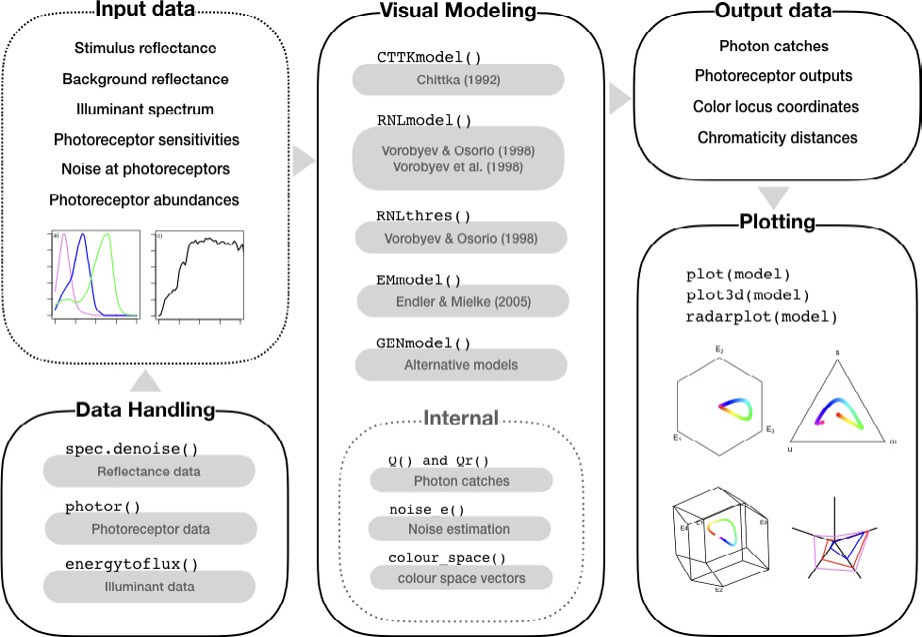
Diagram showing the main functions in colourvision (v2.0) R package. Users provide input data that may be changed by data handling functions. Input data are arguments used by color vision model functions. There are functions to the most commonly used color vision models, and a general function able to generate user‐defined color vision models (GENmodel). These models have been extended to accept any number of photoreceptor types. Some functions are used internally (internal inset) in models but may be of interest for more advanced users. Model functions generate a comprehensive output, which may be visualized into model‐specific color spaces using plotting functions

Colourvision is a package for color vision modeling and presentation of model results (Figure 5). The package implements the general method for *n*‐dimensional models presented above and therefore are able to generate user‐defined color vision models using a simple R function (a model not implemented in colourvision, or a new user‐defined model), which complements other packages and software already available (e.g. pavo, Maia, Eliason, Bitton, Doucet, & Shawkey, 2013). The main advantages of colourvision are (a) the flexibility to build a user‐defined color vision models; (b) extension of all color vision models to any number of photoreceptors; and (c) user‐defined adjustments of color space when changing number of photoreceptors.

Within this unified framework, researchers may easily test variations from current models that may better represent reality. For instance, it is possible to use a tetrachromatic version of Chittka, 1992 color hexagon with same vertex length as in the trichromatic version (in fact with any desired length), instead of a fixed vector length as in Thery and Casas (2002). By extending models to any number of photoreceptor types, colourvision makes it possible, for instance, to model the vision of tentatively pentachromatic organisms (e.g. *Drosophila melanogaster*; Schnaitmann, Garbers, Wachtler, & Tanimoto, 2013), and test model predictions against behavioural data using all models. Furthermore, with the general function to produce user‐defined models, it is possible, for example, to generate a receptor noise limited model that transforms photon catch data by *x*/(*x* + 1) instead of ln (note, however, that these new models have not been validated by behavioural data).

Furthermore, model outputs in *colourvision* can be projected into their chromaticity diagrams using plot functions (Figure 5). For instance, data from a Chittka (1992) model are easily plotted into a hexagonal trapezohedron, which represents the color space boundaries of a tetrachromat in this model. The package also provides additional plotting functions for visualization of photoreceptor inputs and outputs into a radar plot, as well as functions to handle input data (Figure 5).

To provide a quick illustration on the potential application of *colourvision* I used the same setup as in simulation 3 (section 2). However, I randomly sampled 50 flowers to serve as reflectance stimuli, and, instead of the honeybee, I simulated dichromatic, trichromatic, tetracromatic, and pentachromatic animals. I generated all combination of spectral sensitivities curves from 330 to 630 nm, with 30‐nm intervals, and calculated log‐RNL (assuming 0.1 receptor noise to all photoreptors) and CH model outputs. In addition, to test the dependency of Δ*S*‐value to the color space dimensions, I further calculated a CH model, but holding a fixed vertex distance of $\sqrt{3}$, instead of a fixed vector length of 1. I used the maximum mean Δ*S*‐value as a selection rule for the best set of photoreceptors (alternatively one could have applied the number of flowers above a certain threshold; see for instance Chiao, Vorobyev, Cronin, & Osorio, 2000).

**Figure 6:**
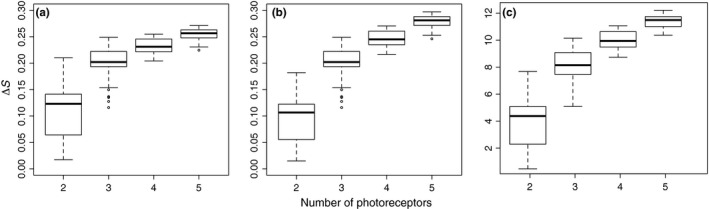
Simulation of flower discrimination (*n* = 50) using combination 2–5 photoreceptors. Δ*S*‐values calculated using (a) the CH model with a fixed vector length, (b) CH model with fixed vertex length, and (c) the log‐RNL model

All three models found the same best set of photoreceptors for di‐, tri‐, tetra‐, and pentachromatic animals: 330 and 420 nm (dichromat), 330, 420, and 570 nm (trichromat), 330, 390, 420, and 570 nm (tetrachromat), and 330, 360, 420, 450, and 570 nm (pentachromat). In addition, distribution of Δ*S*‐values showed an increase in Δ*S*‐values and a reduction in variability as the number of photoreceptor increases (Figure 6). Interestingly, however, the best trichromatic model is as good as most pentachromatic models. Comparison between CH model with fixed vector length and CH with fixed vertex distance shows a similar pattern, but there is a decrease in Δ*S*‐value for <3 photoreceptors and an increase in Δ*S*‐value for >3 photoreceptors (Figure 6).

All calculations and color space figures in this study were performed using the *colourvision* R package (R scripts are available in the Supporting Information Data[Link], [Link], [Link], [Link]), which also illustrate potential package applications. For more detail on how to use colourvision, refer to the user guide vignette (https://cran.r-project.org/web/packages/colourvision/vignettes/colourvision-vignette.html).

## CONFLICTS OF INTEREST

There are no conflicts of interest to declare.

## AUTHOR CONTRIBUTIONS

As a sole author, FMG conducted all steps to produce this manuscript.

## DATA ACCESSIBILITY

Flower reflectance data are publicly accessible via Flower Reflectance Database—FreD (http://www.reflectance.co.uk; Arnold et al., 2010). Simulation R scripts are available in the Supporting Information Appendix S1.
