# Supplementary material for: Color vision models: Some simulations, a general n‐dimensional model, and the colourvision R package
Source: Ecol Evol. 2018 Jul 22;8(16):8159–70. doi: 10.1002/ece3.4288 (PMC6144980; doi:10.1002/ece3.4288)
Supplement: Supplementary file 1 [file ECE3-8-8159-s001.docx]

Figure S1. Basic setup used for colour vision model simulations. (a) Honeybee (*Apis mellifera*) photoreceptor sensitivity curves (data from Peitsch *et al.* 1992 available in

Chittka & Kevan 2005); (b) Achromatic background reflectance spectrum; (c) CIE D65 standard daylight illuminant; and (d) Reflectance spectra generated by a logistic function with midpoints varying from 300 to 700nm, at 5nm intervals. Spectrum colours are arbitrary. In black is shown a reflectance curve with midpoint at 500nm.

Figure S2. Changes from the basic setup used for colour vision model simulations. (a) Ten percentage points removed from the original reflectance spectra with midpoints varying from 300 to 700nm, at 5nm intervals; (b) Achromatic reflectance spectra, with reflectance values from 5% to 95%, at 10 percentage point intervals; (c) Background reflectance spectra calculated from the average reflectance of leaves, leaf litter, grasses and tree bark collected in the Brazilian savanna (data from Gawryszewski and Motta 2012); (d) Reflectance spectra of 859 flowers collected worldwide (data from the Flower Reflectance Database;

Arnold *et al.* 2010).

Figure S3. Flower reflectance spectra (N=858) projected into chromaticity diagrams: Chittka (1992) colour hexagon (CH), Endler & Mielke (2005) colour triangle (EM), and linear and log-linear Receptor Noise Limited models (Linear-RNL and Log-RNL; Vorobyev & Osorio 1998; Vorobyev *et al.* 1998). To facilitate model comparison, point colours correspond to chromaticity distances in the CH chromaticity diagram.

Figure S4. Average photoreceptor sensitivity curves of birds used for tetrachromatic model simulations. Birds with UV λ_max_ cones, and λ_max_ of cones with oil droplets (data from Hart & Vorobyev 2005 available in Endler and Mielke 2005) .

Figure S5. Reflectance spectra generated by a Gaussian function with wavelength of maximum reflectance varying from 300 to 700nm at 5nm intervals. Spectrum colours are arbitrary. In black is shown a reflectance curve with wavelength of maximum reflectance at 500nm.

Figure S6. Ten percentage points added to the reflectance spectra generated by a Gaussian function (Figure S5). Spectrum colours are arbitrary. In black is shown a reflectance curve with wavelength of maximum reflectance at 500nm.

c)

a)

b)

d)

Figure S7. Chromaticity diagrams of the basic setup of tetrachromatic colour vision model simulations: a) Chittka (1992), b) Endler & Mielke (2005) model, and b) linear and c) log-linear Receptor Noise Limited models (Linear-RNL and Log-RNL; Vorobyev & Osorio 1998; Vorobyev et al. 1998). Colours correspond to reflectance spectra from Figure S1.

Figure S8. Tetrachromatic colour vision model basic setup simulations: a) Chittka (1992); b) Endler & Mielke (2005), and b) linear and c) log-linear Receptor Noise Limited models (Linear-RNL and Log-RNL; Vorobyev & Osorio 1998; Vorobyev *et al.* 1998). ΔS-values (top row) and photoreceptor outputs (bottom row) as a function of reflectance spectra with midpoints from 300 to 700nm. Violet, blue, green, and red colours represent UV, short, middle and long λ_max_ photoreceptor types. Vertical lines represent midpoint of maximum ΔS-values.

d)

c)

b)

a)

Figure S9. Chromaticity diagrams with 10 percentage points added to reflectance values: a) Chittka (1992), b) Endler & Mielke (2005) model, and b) linear and c) log-linear Receptor Noise Limited models (Linear-RNL and Log-RNL; Vorobyev & Osorio 1998; Vorobyev et al. 1998). Colours correspond to reflectance spectra from Figure S2a.

Figure S10. Second setup simulations (10 percentage points added to stimulus reflectance spectra) of tetrachromatic colour vision models: a) Chittka (1992); b) Endler & Mielke (2005), and b) linear and c) log-linear Receptor Noise Limited models (Linear-RNL and Log-RNL; Vorobyev & Osorio 1998; Vorobyev *et al.* 1998). ΔS-values (top row) and photoreceptor outputs (bottom row) as a function of reflectance spectra with midpoints from 300 to 700nm. Violet, blue, green, and red colours represent UV, short, middle and long λ_max_ photoreceptor types. Vertical lines represent midpoint of maximum ΔS-values.

Figure S11. Third setup of tetrachromatic colour vision model simulations – achromatic stimulus against chromatic background:: a) Chittka (1992), b) Endler & Mielke (2005), and c) linear and d) log-linear Receptor Noise Limited models (Linear-RNL and Log-RNL; Vorobyev & Osorio 1998; Vorobyev *et al.* 1998). ΔS-values (top row) and photoreceptor outputs (bottom row) as a function of spectra with achromatic reflectance from 5% to 95%. Photoreceptor output values as a function of the same reflectance spectra (bottom row). Violet, blue, green and red colours represent UV, short, middle and long λ_max_ photoreceptor types.

d)

c)

b)

a)

Figure S12. Flower reflectance spectra (N=858) projected into tetrachromatic chromaticity diagrams: a) Chittka (1992), b) Endler & Mielke (2005), and c) linear and d) log-linear Receptor Noise Limited models (Linear-RNL and Log-RNL; Vorobyev & Osorio 1998; Vorobyev *et al.* 1998). To facilitate model comparison, point colours correspond to chromaticity distances in the CH chromaticity diagram.

Figure S13. Chromaticity diagrams of the basic setup of colour vision model simulations with Gaussian reflectance spectra: Chittka (1992) colour hexagon (CH), Endler & Mielke (2005) colour triangle (EM), and linear and log-linear Receptor Noise Limited models (Linear-RNL and Log-RNL; Vorobyev & Osorio 1998; Vorobyev et al. 1998). Colours correspond to reflectance spectra from Figure S5.

Figure S14. Tetrachromatic colour vision model basic setup simulations with Gaussian reflectance curves (Figure S5): a) Chittka (1992); b) Endler & Mielke (2005), and b) linear and c) log-linear Receptor Noise Limited models (Linear-RNL and Log-RNL; Vorobyev & Osorio 1998; Vorobyev *et al.* 1998). ΔS-values (top row) and photoreceptor outputs (bottom row) as a function of reflectance spectra with wavelength of maximum reflectance (λ_peak_) from 300 to 700nm. Violet, blue, and green colours represent short, middle and long λ_max_ photoreceptor types. Vertical lines represent midpoint of maximum ΔS-values.

Figure S15. Chromaticity diagrams of the second simulation – 10 percentage points added to reflectance values (Figure S6): Chittka (1992) colour hexagon (CH), Endler & Mielke (2005) colour triangle (EM), and linear and log-linear Receptor Noise Limited models (Linear-RNL and Log-RNL; Vorobyev & Osorio 1998; Vorobyev *et al.* 1998). Colours correspond to reflectance spectra from Figure S6.

Figure S16. Second setup of colour vision model simulations - 10 percentage points added to stimulus reflectance spectra (Figure S6): Chittka (1992) colour hexagon (CH), Endler & Mielke (2005) colour triangle (EM), and linear and log-linear Receptor Noise Limited models (Linear-RNL and Log-RNL; Vorobyev & Osorio 1998; Vorobyev *et al.* 1998). ΔS-values (top row) and photoreceptor outputs (bottom row) as a function of reflectance spectra with wavelength of maximum reflectance (λ_peak_) from 300 to 700nm. Violet, blue and green colours represent short, middle and long λ_max_ photoreceptor types. Vertical lines represent midpoint of maximum ΔS-values.
