# Supplementary material for: Color vision models: Some simulations, a general n‐dimensional model, and the colourvision R package
Source: Ecol Evol. 2018 Jul 22;8(16):8159–70. doi: 10.1002/ece3.4288 (PMC6144980; doi:10.1002/ece3.4288)
Supplement: Supplementary file 2 [file ECE3-8-8159-s002.docx]

**Supplementary Methods**

1. **Logistic equation used to generate reflectance spectra**

As the stimulus reflectance spectra I generated reflectance curves using a logistic function:

|  | $R\left( \lambda\right)=\frac{L}{1+e^{-k\left( \lambda-\lambda_{mid} \right)}}$ | (Eq. S1) |
| --- | --- | --- |

Where $R$ is the reflectance value at wavelength $\lambda$, $L$ gives the curve maximum reflectance value (%), $k$ gives the steepness of the curve, and $\lambda_{mid}$ is the wavelength (nm) of midpoint. The logistic curve is a typical reflectance curve of many animal colour patches. I used a maximum value of $L=50\%$ reflectance and a steepness of $k=0.04$.

**2. Colour vision model formulae used for simulations**

Colour vision models require a minimum of four parameters for calculations: (1) photoreceptor sensitivity curves, (2) background reflectance spectrum, (3) illuminant spectrum, and (4) the observed object reflectance spectrum (stimulus). In addition, receptor noise limited models require photoreceptor noise for each photoreceptor type. Photoreceptor sensitivity curves are available for several animal taxa. If not available, the sensitivity curves can be estimated using formula based on wavelength at maximum photoreceptor sensitivity ($\lambda_{max}$; Govardovskii *et al.* 2000). Background reflectance can be calculated by measuring the reflectance of materials found in the environment, such as leaves, twigs and tree bark. Alternatively, the background reflectance can be an achromatic spectrum of low reflectance value. The illuminant can be a reference spectrum (e.g. CIE standards), or, ideally, measured directly in the field using an irradiance measurement procedure (Endler 1990; 1993). Reflectance spectra are usually measured using a spectrometer (see Anderson & Prager 2006 for measurement procedures), but it can also be collected using photographic and hyperspectral cameras

(Stevens *et al.* 2007; Chiao *et al.* 2011). All data must cover the same wavelength range as the photoreceptor sensitivity curves (300-700 nm for most cases).

I begin with eq. S2-S4, which are common to all colour vision models presented here. Then calculation for each model is presented in a subtopic. Photoreceptors are grouped by their maximum sensitivity value ($\lambda_{max}$), from shortest to longest $\lambda_{max}$. Honeybees workers (*Apis mellifera*), for instance, have three photoreceptor types with $\lambda_{max}$ at ca. 344nm, 436nm and 544nm (Peitsch *et al.* 1992).

The first step is to calculate the total photon capture ($Q_{i}$) of each photoreceptor type ($i$):

|  | $Q_{i}\left( \lambda\right)=\int_{300}^{700} I\left( \lambda\right)R\left( \lambda\right)C_{i}\left( \lambda\right)d\lambda$ | (Eq. S2) |
| --- | --- | --- |

where $I$ is the illuminant spectrum reaching the observed object, $R$ is the reflectance of the observed object, $C_{i}$ is the photoreceptor sensitivity curve of photoreceptor *i*. The integration is usually done from 300 to 700nm, but this range can be changed depending on the animal of interest. Most mammals, for instance, do not capture photons below 400 nm. The second step is to calculate the photon catch by each photoreceptor (*i*) arising from the background reflectance:

|  | $Q_{Bi}\left( \lambda\right)=\int_{300}^{700} I\left( \lambda\right)R_{B}\left( \lambda\right)C_{i}\left( \lambda\right)d\lambda$ | (Eq. S3) |
| --- | --- | --- |

where $I$ and $C_{i}$ are the same values in eq. S2, and $R_{B}$ is the background reflectance. In practice photon catches are done by summation $Q_{i}\left( \lambda\right)=k\sum_{300}^{700} I\left( \lambda\right){\times R}_{B}\left( \lambda\right)\times C_{i}\left( \lambda\right)$, where *k* is the constant representing the interval between measurements, usually 1 nm. The relative photoreceptor photon catch ($q_{i}$) is then calculated by:

|  | $q_{i}=\frac{Q_{i}}{Q_{Bi}}$ | (Eq. S4) |
| --- | --- | --- |

The rationale behind eq. S3, referred as the von Kries transformation, is that photoreceptors are physiologically adapted to the light coming from the background, and that animals exhibit colour constancy

(Chittka *et al.* 2014).

*2.1 Colour hexagon model*

The colour hexagon model (Chittka 1992) was formulated for hymenopteran vision. However, due to its general form it can, and has been, applied for other taxa. Photoreceptor output ($p$) is given by:

|  | $p_{i}=\frac{q_{i}}{q_{i}+1}$ | (Eq. S5) |
| --- | --- | --- |

This means that photoreceptor outputs ($p$) will vary from 0 to 1, and its value will increase asymptotically to the limit of 1. Photoreceptor output values are then depicted into three vectors evenly distributed. For a trichromat, the resultant of receptor outputs is projected into a plan (chromaticity diagram) using the following formula (Chittka 1992):

|  | $x=\sin60^{\circ}\left( E_{3}-E_{1} \right)$ | (Eq. S6) |
| --- | --- | --- |

|  | $y=E_{2}-\frac{1}{2}\left( E_{1}+E_{3} \right)$ | (Eq. S7) |
| --- | --- | --- |

*2.2 Endler & Mielke (2005) model*

The model is originally the first step for a statistical approach to study bird colouration as whole, not as individual colour patches (Endler & Mielke 2005). The first step is to log-transform relative photon catches:

|  | $p_{i}=\ln\left( q_{i} \right)$ | (Eq. S8) |
| --- | --- | --- |

Then, $f_{i}$ is transformed so that photoreceptor outputs $p_{1}+ p_{2}+\ldots+p_{n} =1$:

|  | $p_{i}=\frac{p_{i}}{p_{1}+ p_{2}+\ldots+p_{n}}$ | (Eq. S9) |
| --- | --- | --- |

Rationale between eq. S9 is that only the relative differences in photoreceptor outputs are used in a colour opponency mechanism. For a trichromat, photoreceptor outputs are transformed into vectors with maximum length = 0.75, to hold the same size as in the original tetrachromatic Endler and Mielke (2005) model. These vectors are projected into a triangular chromaticity diagram by the following formulae (based eq. 1-6, main text):

|  | $V=\frac{3}{4}\left[ \begin{matrix} -\frac{\sqrt{3}}{2} \\ -\frac{1}{2} \end{matrix} \begin{matrix} \frac{\sqrt{3}}{2} \\ -\frac{1}{2} \end{matrix} \begin{matrix} 0 \\ 1 \end{matrix} \right]$ | (Eq. S10) |
| --- | --- | --- |

|  | $\left[ \begin{matrix} x \\ y \end{matrix} \right]=\frac{3}{4}\left[ \begin{matrix} -\frac{\sqrt{3}}{2} \\ -\frac{1}{2} \end{matrix} \begin{matrix} \frac{\sqrt{3}}{2} \\ -\frac{1}{2} \end{matrix} \begin{matrix} 0 \\ 1 \end{matrix} \right]\times\left[ \begin{matrix} p_{1} \\ p_{2} \\ p_{3} \end{matrix} \right]$ | (Eq. S11) |
| --- | --- | --- |
|  | $x=\frac{3\sqrt{3}}{8}\left( p_{2}-p_{1} \right)$ | (Eq. S12) |

|  | $y=\frac{3}{4}\left[ p_{3}-\frac{1}{2}\left( p_{1}+p_{2} \right) \right]$ | (Eq. S13) |
| --- | --- | --- |

*2.3 Receptor noise limited models: linear and log-linear versions*

The receptor noise limited model was developed to predict thresholds of colour vision. One of the assumption is that thresholds are given by noise arising at the receptor channels (Vorobyev & Osorio 1998). The first receptor noise limited model uses a linear relationship between photoreceptor input ($q_{i}$) and output ($p_{i}$) so that (linear version of the receptor noise limited model; Vorobyev & Osorio 1998):

|  | $p_{i}=q_{i}$ | (Eq. S14) |
| --- | --- | --- |

The log-linear version of receptor noise limited model assumes a log-linear relationship between photoreceptor input and output (log-linear version of the receptor noise limited model; (Vorobyev *et al.* 1998):

|  | $p_{i}=\ln\left( q_{i} \right)$ | (Eq. S15) |
| --- | --- | --- |

Eq. S14 can be used when comparing colours that are very similar, otherwise eq. S15 should be used. Subsequently, $p$ values are used to find the colour locus coordinates in a trichromatic chromaticity using the method provided in the main the text:

|  | $V=\left[ \begin{matrix} -\frac{\sqrt{3}}{2} \\ -\frac{1}{2} \end{matrix} \begin{matrix} \frac{\sqrt{3}}{2} \\ -\frac{1}{2} \end{matrix} \begin{matrix} 0 \\ 1 \end{matrix} \right]$ | (Eq. S16) |
| --- | --- | --- |
|  |  |  |
|  | $\vec{p}=\left[ \begin{matrix} p_{1} \\ p_{2} \\ p_{3} \end{matrix} \right]$ | (Eq. S17) |

|  | $R=\left[ \begin{matrix} e_{1}^{2} & 0 & 0 \\ 0 & e_{2}^{2} & 0 \\ 0 & 0 & e_{3}^{2} \end{matrix} \right]$ | (Eq. S18) |
| --- | --- | --- |
|  | $\left[ \begin{matrix} x \\ y \\ z \end{matrix} \right]=\sqrt{\left( VRV^{T} \right)^{-1}}Vp$ | (Eq. S19) |

where $e_{i}$ is the receptor noise of each photoreceptor, from shortest to longest wavelength. To date few species had their receptor noise ($e_{i}$) measured directly (Vorobyev & Osorio 1998). In lack of a direct measurement, $e_{i}$ can be estimated by the relative abundance of photoreceptor types in the retina, and a measurement of a single photoreceptor noise-to-signal ratio (Vorobyev *et al.* 1998; Vorobyev & Osorio 1998):

|  | $e_{i}=\frac{\nu}{\sqrt{\eta_{i}}}$ | (Eq. S20) |
| --- | --- | --- |

Where $\nu$ is the noise-to-signal ratio of a single photoreceptor, and $\eta_{i}$ is the relative abundance of photoreceptor $i$ in the retina. Alternatively, $e_{i}$ may be intensity dependent, where noise depends on the photon catch given by eq. S2

(see Vorobyev *et al.* 1998 and Renoult *et al.* 2017). Eq. S20 is usually valid in high light intensities, but in dim light conditions receptor noise becomes dependent on the photon catches (Vorobyev *et al.* 1998; Vorobyev & Osorio 1998).

**3. Methods for tetrachromatic colour vision models**

*3.1. Colour hexagon model for tetrachromats*

The tetrachromat version of Chittka (1992) colour hexagon was derived by Thery & Casas (2002). The photoreceptor outputs are calculated in the same way as the trichromatic version. Then, these values are projected into a tridimensional colour space, a hexagonal trapezohedron, by the following formulae (Thery & Casas 2002):

|  | $x=\frac{\sqrt{2}\sqrt{3}}{3}\left( p_{3}-p_{4} \right)$ | (Eq. S21) |
| --- | --- | --- |

|  | $y=p_{1}-\frac{1}{3}\left( p_{2}+p_{3}+p_{4} \right)$ | (Eq. S22) |
| --- | --- | --- |

|  | $z=\frac{2\sqrt{2}}{3}\left[ \frac{1}{2}\left( p_{3}+p_{4} \right)-p_{2} \right]$ | (Eq. S23) |
| --- | --- | --- |

*3.2. Endler and Mielke (2005) model*

Model calculation follow the same steps as in the trichromatic version, but with four photoreceptor outputs. Then, $p_{i}$ is transformed so that photoreceptor outputs $u+s+m+l=1$:

|  | $u=\frac{p_{1}}{p_{1}+p_{2}+p_{3}+p_{4}}$ | (Eq. S24) |
| --- | --- | --- |

|  | $s=\frac{p_{2}}{p_{1}+p_{2}+p_{3}+p_{4}}$ | (Eq. S25) |
| --- | --- | --- |

|  | $m=\frac{p_{3}}{p_{1}+p_{2}+p_{3}+p_{4}}$ | (Eq. S26) |
| --- | --- | --- |

|  | $l=\frac{p_{4}}{p_{1}+p_{2}+p_{3}+p_{4}}$ | (Eq. S27) |
| --- | --- | --- |

Photoreceptor outputs are then used find colour locus into a tridimensional colour space (tetrahedron) by the following formulae:

|  | $x=\sqrt{\frac{3}{2}}\left( \frac{1-2s-m-u}{2} \right)$ | (Eq. S28) |
| --- | --- | --- |

|  | $y=\frac{-1+3m+u}{2\sqrt{2}}$ | (Eq. S29) |
| --- | --- | --- |

|  | $z=u-\frac{1}{4}$ | (Eq. S30) |
| --- | --- | --- |

*3.3. Receptor noise limited models: linear and log-linear versions*

For linear-RNL and log-RNL models, photoreceptor outputs and photoreceptor noise of each photoreceptor type are used to find colour locus coordinates into chromaticity diagram using the following formulae provided in the main text:

|  | $V=\left[ \begin{matrix} {-\sqrt{2}\sqrt{3}}/3 & {\sqrt{2}\sqrt{3}}/3 & 0 \\ {-\sqrt{2}}/3 & {-\sqrt{2}}/3 & {2\sqrt{2}}/3 \\ {-1}/3 & {-1}/3 & {-1}/3 \end{matrix} \begin{matrix} 0 \\ 0 \\ 1 \end{matrix} \right]$ | (Eq. S31) |
| --- | --- | --- |
|  |  |  |
|  | $\vec{p}=\left[ \begin{aligned} \begin{matrix} p_{1} \\ p_{2} \\ p_{3} \end{matrix} \\ p_{4} \end{aligned} \right]$ | (Eq. S32) |

|  | $R=\left[ \begin{aligned} \begin{matrix} e_{1}^{2} & 0 \\ 0 & e_{2}^{2} \end{matrix} \begin{matrix} 0 & 0 \\ 0 & 0 \end{matrix} \\ \begin{matrix} 0 & 0 \\ 0 & 0 \end{matrix} \begin{matrix} e_{3}^{2} & 0 \\ 0 & e_{4}^{2} \end{matrix} \end{aligned} \right]$ | (Eq. S33) |
| --- | --- | --- |
|  | $\left[ \begin{matrix} x \\ y \\ z \end{matrix} \right]=\sqrt{\left( VRV^{T} \right)^{-1}}Vp$ | (Eq. S34) |

where $e_{i}$ is the receptor noise of each photoreceptor, from shortest to longest wavelength.

*3.4. Distance between colour loci*

Chromaticity distance between pair of reflectance spectra ($a$ and $b$) is found by calculating the Euclidian distance between their colour loci in the colour space:

|  | $\Delta S=\sqrt{\left( x_{a}-x_{b} \right)^{2}+\left( y_{a}-y_{b} \right)^{2}+\left( z_{a}-z_{b} \right)^{2}}$ | (Eq. S35) |
| --- | --- | --- |

By definition background reflectance lays at the centre of the background ($x=0, y=0)$. Therefore, the distance of the observed object against the background is given by:

|  | $\Delta S=\sqrt{x^{2}+y^{2}+z^{2}}$ | (Eq. S36) |
| --- | --- | --- |

In the original noise receptor model (Vorobyev and Osorio 1998) $\Delta S$ between pair of reflectance spectra ($a$ and $b$) is calculated directly, without finding colour locus coordinates:

$$\Delta S=\sqrt{\frac{\begin{aligned} {\left( e_{1}e_{2} \right)^{2}\left( {\Delta p}_{4}-{\Delta p}_{3} \right)}^{2}+{\left( e_{1}e_{3} \right)^{2}\left( {\Delta p}_{4}-{\Delta p}_{2} \right)}^{2}+{\left( e_{1}e_{4} \right)^{2}\left( {\Delta p}_{3}-{\Delta p}_{2} \right)}^{2}+ \\ {\left( e_{2}e_{3} \right)^{2}\left( {\Delta p}_{4}-{\Delta p}_{1} \right)}^{2}+{\left( e_{2}e_{4} \right)^{2}\left( {\Delta p}_{3}-{\Delta p}_{1} \right)}^{2}+{\left( e_{3}e_{4} \right)^{2}\left( {\Delta p}_{2}-{\Delta p}_{1} \right)}^{2} \end{aligned}}{\left( e_{1}e_{2}e_{3} \right)^{2}+\left( e_{1}e_{3}e_{4} \right)^{2}+\left( {e_{1}e}_{2}e_{4} \right)^{2}+\left( {e_{2}e}_{3}e_{4} \right)^{2}}}$$

|  |  | (Eq. S37) |
| --- | --- | --- |

Where $\Delta p_{i}$ is the difference between photoreceptor *i* output for the reflectance spectrum $a$ and $b$ ($\Delta p_{i}=p_{a_{i}}-p_{b_{i}}$). Using eq. S37 will give the same value as calculating $\Delta S$ using eq. S31-34 and then eq. S35.

**4. Simulations with tetrachromatic vision**

Model simulation parameters were the same as in the trichromatic simulation, except that instead of honeybee photoreceptors, I used the average photoreceptor sensitivity curves of birds (only birds with UV λ_max_ cones; data from Hart & Vorobyev 2005 available in Endler & Mielke 2005; Figure S4). I estimated receptor noise using eq. S201, with a ratio of 1:2:2:4 photoreceptor types in the retina (from shortest to longest lambda-max; *Leiothrix lutea*) and a noise-to-signal ratio of 0.1; data available in Vorobyev & Osorio 1998; Vorobyev *et al.* 1998). Results are presented in Figures S7-S12.

**5. Simulations using Gaussian reflectance curves**

Model simulation parameters were the same as in the original simulations, except that instead of a logistic function, I used a Gaussian function to generate stimulus reflectance spectra:

|  | $R(\lambda)=ae^{-\frac{{{(\lambda-\lambda}_{p})}^{2}}{2b^{2}}}$ | (Eq. S38) |
| --- | --- | --- |

Where $R$ is the reflectance value at wavelength $\lambda$, $a$ gives the curve maximum reflectance value (%), $b$ controls the width of the curve, and $\lambda_{p}$ is the wavelength (nm) of maximum reflectance. I used a maximum value of $a=50\%$ reflectance, and a width of $b=0.04$. I generated curves with wavelength of maximum reflectance varying from 300 to 700 nm with 5 nm intervals, in a total of 81 reflectance spectra (Figures S5 and S6). Results are presented in Figures S13-S16.

**6. References**

Anderson, S. & Prager, M. (2006). Quantifying Colors. *Bird Coloration: Volume 1, Mechanisms and Measurements* (eds. G. E. Hill & K. J. McGraw), pp. 41-89. Harvard University Press, Cambridge, MA, USA.

Chiao, C.-C., Wickiser, J.K., Allen, J.J., Genter, B. & Hanlon, R.T. (2011). Hyperspectral imaging of cuttlefish camouflage indicates good color match in the eyes of fish predators. *Proceedings of the National Academy of Sciences*, **108**, 9148–9153.

Endler, J.A. & Mielke, P. (2005). Comparing entire colour patterns as birds see them. *Biological Journal Of The Linnean Society*, **86**, 405–431.

Chittka, L. (1992). The colour hexagon: a chromaticity diagram based on photoreceptor excitations as a generalized representation of colour opponency. *Journal of Comparative Physiology A: Sensory, Neural, and Behavioral Physiology*, **170**, 533–543.

Chittka, L., Faruq, S., Skorupski, P. & Werner, A. (2014). Colour constancy in insects. *Journal of Comparative Physiology A: Sensory, Neural, and Behavioral Physiology*, **200**, 435–448.

Endler, J.A. (1990). On the measurement and classification of colour in studies of animal colour patterns. *Biological Journal Of The Linnean Society*, **41**, 315–352.

Endler, J.A. (1993). The color of light in forests and its implications. *Ecological Monographs*, **63**, 1–27.

Endler, J.A. & Mielke, P. (2005). Comparing entire colour patterns as birds see them. *Biological Journal Of The Linnean Society*, **86**, 405–431.

Govardovskii, V.I., Fyhrquist, N., Reuter, T., Kuzmin, D.G. & Donner, K. (2000). In search of the visual pigment template. *Visual Neuroscience*, **17**, 509–528.

Hart, N.S. & Vorobyev, M. (2005). Modelling oil droplet absorption spectra and spectral sensitivities of bird cone photoreceptors. *Journal of Comparative Physiology A: Sensory, Neural, and Behavioral Physiology*, **191**, 381–392.

Peitsch, D., Fietz, A., Hertel, H., de Souza, J., Ventura, D.F. & Menzel, R. (1992). The spectral input systems of hymenopteran insects and their receptor-based color-vision. *Journal of Comparative Physiology A: Sensory, Neural, and Behavioral Physiology*, **170**, 23–40.

Renoult, J.P., Kelber, A. & Schaefer, H.M. (2017). Colour spaces in ecology and evolutionary biology. *Biological Reviews Of The Cambridge Philosophical Society*, **92**, 292–315.

Stevens, M., Párraga, C.A., Cuthill, I.C., Partridge, J.C. & Troscianko, T. (2007). Using digital photography to study animal coloration. *Biological Journal of The Linnean Society*, **90**, 211–237.

Thery, M. & Casas, J. (2002). Predator and prey views of spider camouflage. *Nature*, **415**, 133–133.

Vorobyev, M. & Osorio, D. (1998). Receptor noise as a determinant of colour thresholds. *Proceedings of the Royal Society B: Biological Sciences*, **265**, 351–358.

Vorobyev, M., Osorio, D., Bennett, A.T.D., Marshall, N.J. & Cuthill, I.C. (1998). Tetrachromacy, oil droplets and bird plumage colours. *Journal of Comparative Physiology A: Sensory, Neural, and Behavioral Physiology*, **183**, 621–633.
